# Twofold role of calcined hydrotalcites in the degradation of methyl parathion pesticide

**DOI:** 10.3762/bjnano.2.11

**Published:** 2011-02-09

**Authors:** Alvaro Sampieri, Geolar Fetter, María Elena Villafuerte-Castrejon, Adriana Tejeda-Cruz, Pedro Bosch

**Affiliations:** 1 Benemérita Universidad Autónoma de Puebla, Facultad de Ingeniería Química, Av. San Claudio, Ciudad Universitaria, 72570, Puebla, PUE, Mexico. Phone: (+52) 22222-95500 ext. 7250; 2 Benemérita Universidad Autónoma de Puebla, Facultad de Ciencias Químicas, Av. San Claudio, Ciudad Universitaria, 72570, Puebla, PUE, Mexico; 3 Universidad Nacional Autónoma de México, Instituto de Investigaciones en Materiales, A.P. 70360, Ciudad Universitaria, 04510 México, D.F., Mexico

**Keywords:** basicity, hydrotalcite, methyl parathion degradation, mixed oxide, organophosphate, pesticide, water pollution

## Abstract

Methyl parathion (MP) is a very toxic organophosphate pesticide used as a non-systematic insecticide and acaricide on many corps. As MP and its by-products are highly toxic, they have to be retained to avoid pollution of rivers and lakes. Highly efficient sorbents are hydrotalcites (HTs) (or anionic clays). We have correlated the degradation of an aqueous solution of MP at room temperature, with the basicity of the adsorbing materials. It was found that the metal composition of hydrotalcites determines both the surface electronic properties (basic or acidic) and the sorption capacity. Depending on the basic strength, some calcined hydrotalcites can catalyze the transformation of MP to *p-*nitrophenol (*p*-NP) and retain its by-products. Such a process has the advantage of being able to be carried out at room temperature and at the pH of the pesticide solution.

## Introduction

MP, an organophosphate, has been extensively used as pesticide since the 1970’s instead of chlorinated hydrocarbons (e.g., DDT). This pesticide is persistent and very toxic to humans and animals [1], even at low concentrations. The symptoms of MP poisoning include, i.a., reduction of body weight, anemia and decreased brain acetylcholinesterase activity [2–3]. Although the Basel, Rotterdam and Stockholm Conventions [4] have forbidden hazardous pesticides including MP, this pesticide is still used for many corps. MP and its degradation products have to be retained and/or decomposed in aqueous solutions. Studies concerning the surface modification of cationic clays and hydrotalcites with organic ions, as pesticide sorbents, have been reviewed by Cornejo et al. [5]. For instance, MP undergoes degradation when it is sorbed on a bentonite (cationic clay) [6–8]. Moreover, the bentonite sorption capacity may be increased if the clay is exchanged with cetyltrimethylammonium bromide (CTAB). Indeed, the hydrophobic character of the pesticide and the organoclay enhances the retention through two mechanisms; either MP may be reduced directly to *p*-NP (degradation) or via an intermediate molecule (isomerization). At pH 9 or 10, the second mechanism is favored (65%) due to the basic catalytic features of the clay hydroxyl ions (OH). Although MP can decompose to give simple molecules, such as SO_2_, CO_2_, CO and NO_2_, these products can also cause several environmental problems.

Thus anionic clays, or simply hydrotalcites (HTs), should be good candidates for MP degradation at room temperature. HTs are layered compounds with basic properties whose chemical formula is: [M^2+^_1−_*_x_*M^3+^*_x_*(OH)_2_](A*^m^*^−^)*_x_*_/_*_m_*·*n*H_2_O [9–12] where M^2+^ may be replaced by trivalent metal cations, M^3+^, which give rise to positively charged layers. This charge is neutralized by A*^m^*^−^, a compensating anion with charge *m* such as CO_3_^2−^, SO_4_^2−^, Cl^−^, OH^−^ or NO_3_^−^ etc. The metal ratio (*x* = M^3+^/(M^3+^ + M^2+^)), and the synthesis procedure (sol–gel, ultrasound or microwave irradiation, among others) determine the properties of the HT-like compounds [9–17]. HTs are versatile lamellar compounds which may exchange anions. Carbonates are always preferred and it is difficult to inhibit the formation of carbonated HTs [18–19]. The layered structure collapses at temperatures between 200–400 °C due to dehydration, dehydroxylation and decarboxylation. The resulting mixed oxides are usually solid solutions with a periclase-like structure [20], which in presence of an anion solution leads to the reconstruction of the hydrotalcite. This process is known as “memory effect”. For example, for the retention of iodide a carbonated hydrotalcite must be thermally treated to eliminate CO_3_^2−^ to obtain the metallic oxides which are the precursors for the reconstruction of HTs with iodide as interlayered anion [21]. HTs can also trap organic anions to give hybrid materials, which have been recently studied as materials for drug delivery [22] or green pesticides [23].

As the basic properties of HTs depend on the metal composition and the M^3+^/(M^3+^ + M^2+^) ratio, [24–26], in this work we prepared HTs with different M^2+^ composition maintaining a molar ratio of M^2+^/Al^3+^ of 2. We correlated the influence of the M^2+^ metal and the basic properties of calcined HTs with the degradation of MP. Whilst the degradation of MP is not well understood, we have studied the effect of the solid basic sites through a systematic modification of the basic centers in the adsorbing material. From this, an ideal configuration of basic sites can be proposed for the effective degradation of MP.

## Results

**Hydrotalcite (HT) characterization:** The diffraction patterns of the Mg–Al, Zn–Al, Ni–Al dried HTs display the characteristic peaks of double layered hydroxides (see Supporting Information File 1 for the experimental procedure for preparation and Supporting Information File 2 for the XRD diffractograms). The interlayer distances (d_(003)_ ≈ 0.78 nm), in all the XRD diffractograms, is typical of carbonated HTs. After calcination at 500 °C, the HTs show X-ray patterns with marked differences (Supporting Information File 2 for XRD diffractograms). This process leads to the formation of mixed oxides whose crystallite size depends on their crystallization rate [9–12,27–28]. For instance, NiO and MgO oxides, from Mg–Al and Ni–Al calcined HTs, have a highly disperse periclase-like structure (MgO) and is usually formed between 450 and 500 °C. Aluminum is then incorporated into the periclase framework. However, in Zn–Al, the Zn species crystallize more rapidly and the only detected compound is ZnO as wurtzite, without any aluminum atoms into the framework. Aluminum is not observed by XRD since it forms a microcrystalline oxide which is undetected by this technique. Mixed oxides should be more efficient for the decomposition of MP than the dried HTs, as they can present interesting acidic–basic properties [26]. Furthermore, mixed oxides should have the ability to regenerate the layered structures of HTs by the “memory effect” in the presence of anions released during MP degradation.

**Figure 1:**
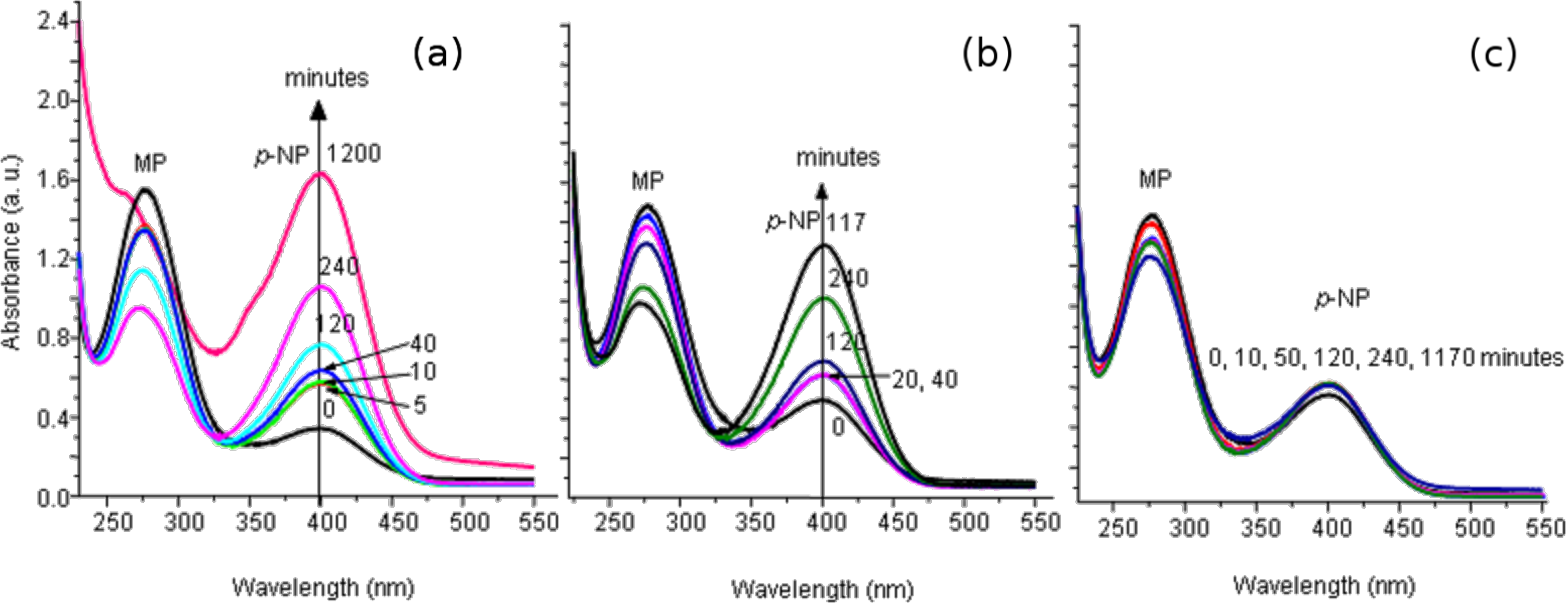
UV–vis spectra of MP solution after being in contact with Mg–Al (a), Zn–Al (b) and Ni–Al. (c) hydrotalcite oxides, MP: methyl parathion, *p*-NP = *p*-nitrophenol.

**Figure 2:**
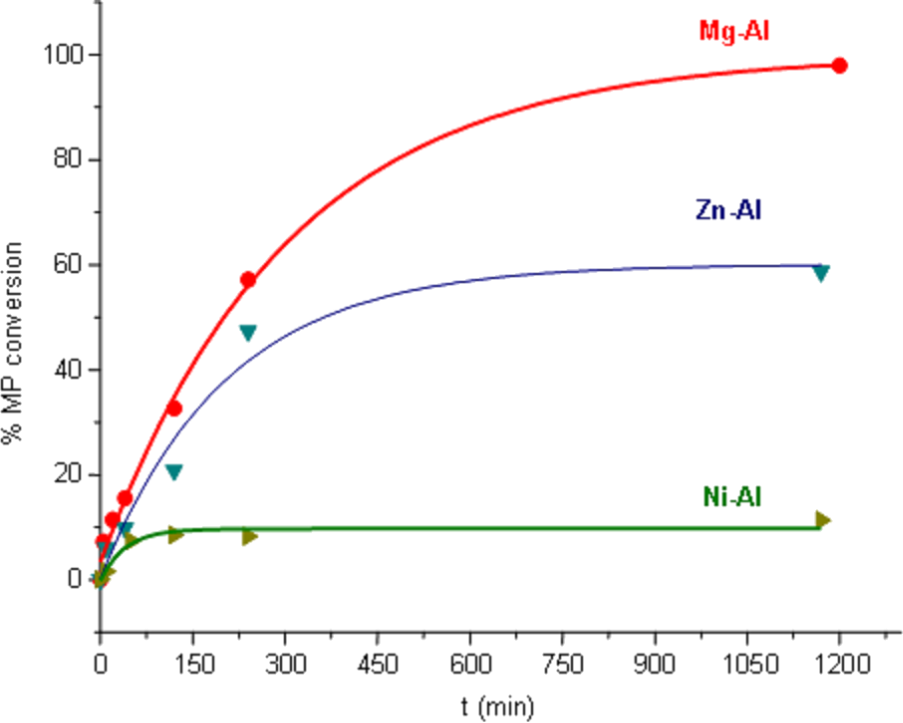
Conversion of MP to *p*-NP from waste solutions treating with hydrotalcite mixed oxides: Mg–Al, Zn–Al and Ni–Al.

**Degradation of MP:** Figure 1 shows the UV–vis absorption spectra of the solution after stirring MP with the Mg–Al, Zn–Al and Ni–Al mixed oxides (see Supporting Information File 1 for full experimental data). Initially (time = 0 minutes), MP is identified by the intense and broad peak at 270 nm, whilst the small (unexpected) peak appears at 410 nm is attributed to *p*-NP. At room temperature, the instability of MP in aqueous solution is such that it decomposes by hydrolysis to *p*-NP. Despite of this, we were able to determine and follow the degradation of MP on the mixed oxides. In Mg–Al, the first peak (MP) decreases overtime (Figure 1a) whereas the second (*p*-NP) increases. At 1200 minutes, MP degradation to *p*-NP is complete. By contrast, the Zn–Al sample was less effective (Figure 1b) and after 1170 minutes of the sorption process MP still remained in the waste solution as *p*-NP. The result using Ni–Al mixed oxide as sorbent was surprising, as it did not promote the degradation of MP (Figure 1c). Figure 2 shows the MP degradation performance to *p*-NP of Mg–Al, Zn–Al and Ni–Al hydrotalcite oxides where the conversions were 100%, 41% and ~0%, respectively.

**Figure 3:**
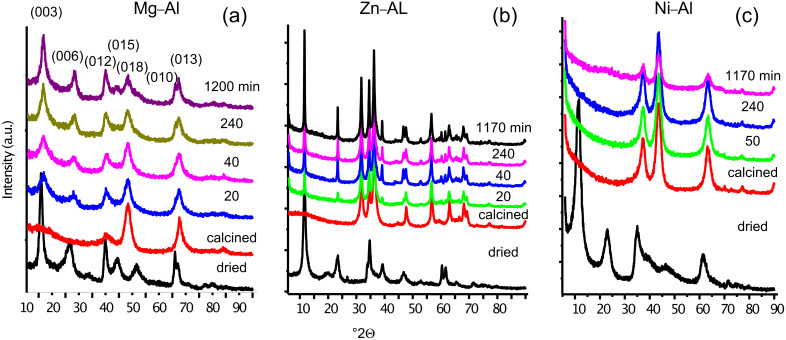
X-ray diffractograms corresponding to Mg–Al (a), Zn–Al (b) and Ni-Al (c) calcined hydrotalcite after being in contact at different times with a MP solution (XRD patterns of both dried and calcined HTs are also presented).

After the degradation of MP, the resulting solids were recovered, dried at room temperature and characterized by X-ray diffraction (Figure 3). The diffractograms of the Mg–Al samples, Figure 3a (recorded after different degradation times) show that the hydrotalcite has been regenerated without any other crystalline compound. Such structure regeneration is intimately related to the increasing MP conversion (Figure 2). The HT layered structure has been built with the anions present in the pesticide solution, not only with carbonates but also with other anionic entities such as phosphate, nitrate and sulfate. However, the X-ray diffraction patterns of Zn–Al (Figure 3b) show that the hydrotalcite structure is only partly recovered, which is in accord with the MP degradation behavior. As expected, the X-ray diffraction patterns of the recovered Ni–Al samples (Figure 3c) are similar to that of the calcined sample, there was no HT reconstruction, as no MP decomposition was observed.

## Discussion

**Figure 4:**
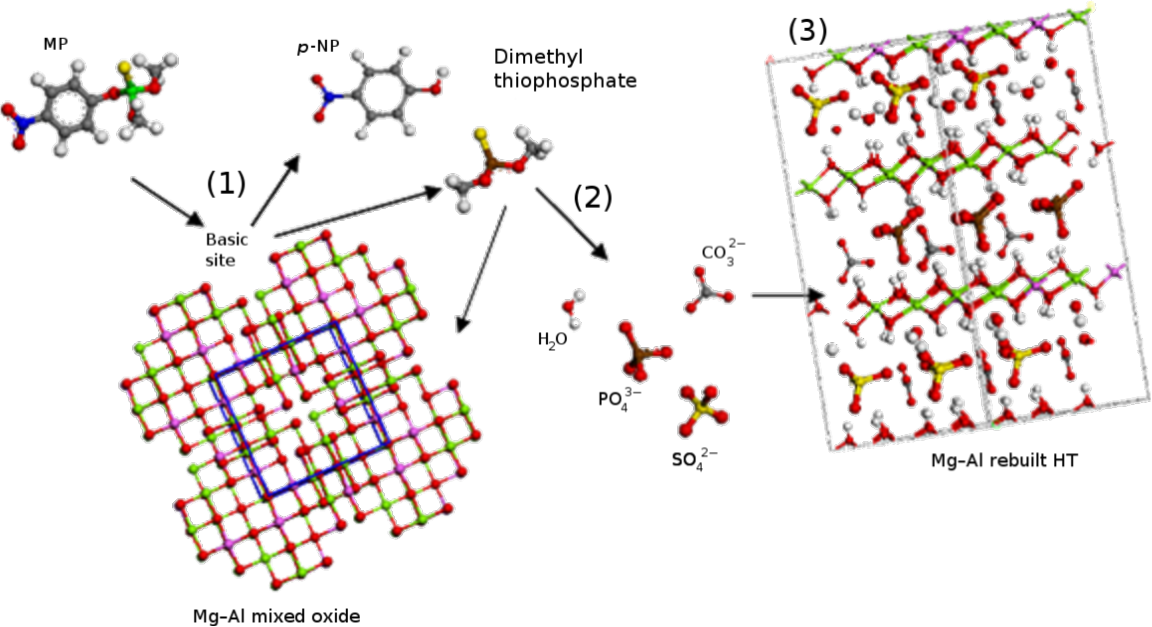
Schematic representation of MP degradation on a basic site of Mg–Al calcined HT (1), followed by the anions released (2) and the HT structure regeneration (3). Red: O, green: Mg, purple: Al, brown: P, white: H, yellow: S, blue: N, gray: C.

Our results may be summarized as follows: Only the recovery of hydrotalcite indicates the degradation of MP and only certain compositions of hydrotalcite show this behavior: Mg–Al and Zn–Al oxides both promote MP degradation to *p*-NH whereas Ni–Al does not. Thus, M^2+^ ions play a significant role in the degradation of MP in aqueous solution. We have recently reported that the acid–base properties and the electronegativity of M^2+^ of some calcined HTs determine the efficiency in CO_2_ adsorption [25–26]. Calcined Ni^2+^-HT presents a higher electronegativity than Mg^2+^-HT. Hence, the Ni–Al mixed oxides have weak basic sites which are not strong enough to promote MP degradation. By contrast, the basic sites of the Mg–Al sample are very active in the MP decomposition. Therefore, the M^2+^ composition of the three samples used determines the layer charge and thus the strength of the basic sites. Although, the large MP molecules cannot reach the sites located in the interlayer space of HTs, the reaction may occur on the sites present at the lamella edges (Figure 4). The MP molecule can be considered as being constructed from a basic fraction (*p*-nitrophenol) and an acidic fraction (dimethyl thiophosphate): The degradation reaction occurs through the MP acid fraction and the basic sites of the hydrotalcite oxides.

The second part of the MP reaction (acid fraction) involves partial decomposition into PO_4_^3−^, SO_4_^2−^, NO_3_^−^ and CO_3_^2−^ ions which enable HT reconstruction through a memory effect mechanism (Figure 4). However, if the resulting species are larger (i.e., dimethyl thiophosphate), they should be sorbed on the edges of the layers. According to the X-ray diffraction patterns, only the Mg–Al sample is fully regenerated into a HT, whereas the Zn–Al one is only partially regenerated. HT reconstruction from Ni–Al oxides does not occur since MP is not decomposed since this material has no basic active sites.

## Conclusion

In this work, the basic character of hydrotalcite oxides (M^2+^/Al^3+^) is reported for the first time to be essential for MP degradation. The resulting compounds are (*p*-NP) and other inorganic (sulfur and phosphorus containing compounds etc.) substances. The degradation of MP with hydrotalcite oxides is a heterogeneization of a homogeneous catalysis mechanism that depends on the basic strength of calcined HTs. The M^2+^/Al^3+^ composition of HT is a key parameter in MP degradation and increases in the following order: Ni–Al<<<Zn–Al<Mg–Al. With degradation, anions are produced and recovered as they participate in the rebuilding of hydrotalcite through a memory effect mechanism. For instance, the role of Mg–Al or Zn–Al HTs is twofold, on the one hand as a catalyst (mixed oxide) accelerating the MP degradation and on the other hand as an anion sorbent through layered structure regeneration. Calcined HTs are promising nanolayer materials for the effective cleansing of water contaminated by organic pesticides.

## Supporting Information

File 1 Experimental section.

File 2 X-ray diffractograms of dried and calcined hydrotalcites.
