# Supplementary material for: Twofold role of calcined hydrotalcites in the degradation of methyl parathion pesticide
Source: Beilstein J Nanotechnol. 2011 Feb 9;2:99–103. doi: 10.3762/bjnano.2.11 (PMC3148045; doi:10.3762/bjnano.2.11)
Supplement: File 1 — Experimental section. [file Beilstein_J_Nanotechnol-02-99-s001.pdf]

Supporting Information File 1

for

Twofold role of calcined hydrotalcites in the degradation of methyl parathion pesticide

Alvaro Sampieri[1*], Geolar Fetter[2], María Elena Villafuerte-Castrejon[3], Adriana Tejeda-Cruz[3] and Pedro Bosch[3]

Address: [1]Benemérita Universidad Autónoma de Puebla, Facultad de Ingeniería Química, Av. San Claudio, Ciudad Universitaria, 72570, Puebla, PUE, Mexico. Phone: (+52) 22222-95500 ext. 7250; [2]Benemérita Universidad Autónoma de Puebla, Facultad de Ciencias Químicas, Av. San Claudio, Ciudad Universitaria, 72570, Puebla, PUE, Mexico and [3]Universidad Nacional Autónoma de México, Instituto de Investigaciones en Materiales, A.P. 70360, Ciudad Universitaria, 04510 México, D.F., Mexico

Email: Alvaro Sampieri* - asamcr@yahoo.com; Geolar Fetter - geolarfetter@yahoo.com.mx; María Elena Villafuerte-Castrejon - mevc@servidor.unam.mx; Adriana Tejeda-Cruz - tejeda@iim.unam.mx; Pedro Bosch - croqcroq@hotmail.com

* Corresponding author

1. Experimental

1.1. Hydrotalcite synthesis: Binary hydrotalcites (HTs) containing Mg^{2+}–Al^{3+}, Zn^{2+}–Al^{3+}, or Ni^{2+}–Al^{3+} with a M^{2+}/Al^{3+} molar ratio of 2/1 were synthesized by coprecipitation [1]. An aqueous solution containing $M^{2+}(NO_3)_2$, (98% Aldrich) and $Al(NO_3)_3$, (99% Aldrich) as

hydrated salts, with a concentration of 2.5 M, and a solution of NaOH (2 M) were added dropwise into a flask at room temperature. An aqueous solution of sodium carbonate (1 M, Na_2CO_3) was also added to the mixture. The rate of addition of the solutions was adjusted in order to obtain a constant pH of 11.5. The mixture was then heated in an autoclave at 80 °C for 24 h. The solids were recovered by decantation and washed with distilled water until the pH of the washing water was 10. The material was then dried in an oven at 70 °C. All HTs were calcined at 500 °C in a furnace for 4 h in order to obtain the mixed oxides.

1.2. Methyl parathion sorption: An aqueous of MP with a concentration of 250 µmol L^{-1} was prepared and stirred for 24 h until complete dissolution. The pH of the slightly yellow solution was 7.0–7.5. Then 100 mg of mixed oxides, from calcined HT, were placed in a flask containing the MP solution. The mixture was stirred for 0, 5, 10, 40, 120, 1170 or 1200 min at room temperature. The mixed solutions were centrifuged to separate the solid and their absorption spectra were analyzed by UV–vis spectroscopy in the wavelength range from 200 to 550 nm.

1.3. X-ray diffraction: A Bruker-axs D8-advance diffractometer coupled to a copper anode X-ray tube was used to investigate the compounds present in the powdered samples. A diffracted beam monochromator selected the Kα radiation.

1.4. UV–vis spectroscopy: Samples were removed periodically and the degradation ratio of methyl parathion monitored by UV–vis spectroscopy (UNICAM 300 UV–Vis spectrometer).

References

1. Cavani, F.; Trifirò, F.; Vaccari, A. *Catal. Today* **1991,** *11*, 173–301. doi:10.1016/0920-5861(91)80068-K
