# Supplementary material for: Twofold role of calcined hydrotalcites in the degradation of methyl parathion pesticide
Source: Beilstein J Nanotechnol. 2011 Feb 9;2:99–103. doi: 10.3762/bjnano.2.11 (PMC3148045; doi:10.3762/bjnano.2.11)

Supporting Information File 2

for

Twofold role of calcined hydrotalcites in the degradation of methyl parathion pesticide

Alvaro Sampieri[1*], Geolar Fetter[2], María Elena Villafuerte-Castrejon[3], Adriana Tejeda-Cruz[3] and Pedro Bosch[3]

Address: [1]Benemérita Universidad Autónoma de Puebla, Facultad de Ingeniería Química, Av. San Claudio, Ciudad Universitaria, 72570, Puebla, PUE, Mexico. Phone: (+52) 22222-95500 ext. 7250; [2]Benemérita Universidad Autónoma de Puebla, Facultad de Ciencias Químicas, Av. San Claudio, Ciudad Universitaria, 72570, Puebla, PUE, Mexico and [3]Universidad Nacional Autónoma de México, Instituto de Investigaciones en Materiales, A.P. 70360, Ciudad Universitaria, 04510 México, D.F., Mexico

Email: Alvaro Sampieri* - asamcr@yahoo.com; Geolar Fetter - geolarfetter@yahoo.com.mx; María Elena Villafuerte-Castrejon - mevc@servidor.unam.mx; Adriana Tejeda-Cruz - tejeda@iim.unam.mx; Pedro Bosch - croqcroq@hotmail.com

* Corresponding author

X-ray diffractograms of dried and calcined hydrotalcites

The X-ray diffractograms of dried (70 °) and calcined (500 °C) hydrotalcites, respectively, are shown in the following figures:

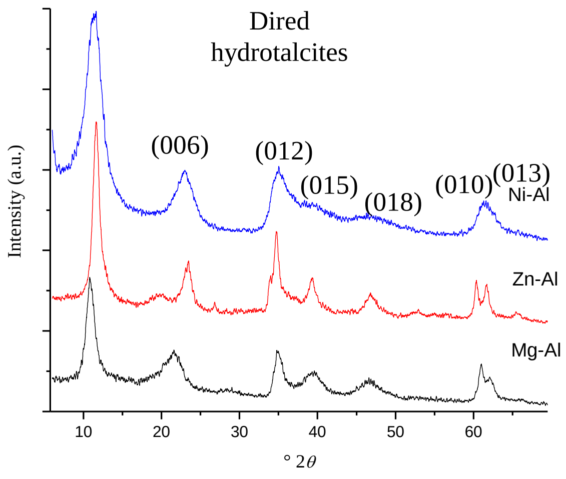

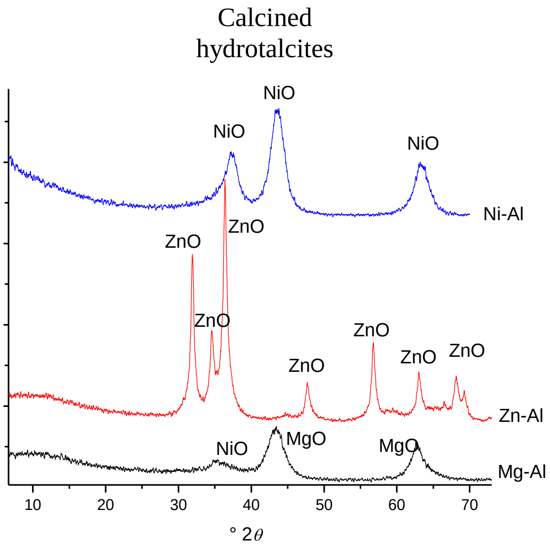

Supplement: File 2 — X-ray diffractograms of dried and calcined hydrotalcites. [file Beilstein_J_Nanotechnol-02-99-s002.pdf]
